# Developing and validating a risk prediction model for conversion to type 2 diabetes mellitus in women with a history of gestational diabetes mellitus: protocol for a population-based, data-linkage study

**DOI:** 10.1136/bmjopen-2025-106052

**Published:** 2025-09-14

**Authors:** Vincent Versace, Douglas Boyle, Edward Janus, James Dunbar, Tesfaye R Feyissa, Yitayeh Belsti, Peta Trinder, Joanne Enticott, Brett Sutton, Jane Speight, Jacqueline Boyle, Shamil Deshan Cooray, Hannah Beks, Sharleen O’Reilly, Kevin Mc Namara, Alice R Rumbold, Siew Lim, Zanfina Ademi, Helena J Teede

**Affiliations:** 1 Deakin Rural Health, School of Medicine, Deakin University, Warrnambool, Victoria, Australia; 2 Geohealth Laboratory, Department of Population Health, Dasman Diabetes Institute, Kuwait City, Al Asimah Governate, Kuwait; 3 Health & Biomedical Research Information Technology Unit (HaBIC R2), Department of General Practice and Primary Care, Faculty of Medicine, Dentistry & Health Sciences, University of Melbourne, Parkville, Victoria, Australia; 4 Western Health Chronic Disease Alliance and Department of Medicine, Western Health—Melbourne Medical School, University of Melbourne, Parkville, Victoria, Australia; 5 Deakin University, School of Medicine, Warrnambool Campus, Warrnambool, Victoria, Australia; 6 Monash Centre for Health Research and Implementation, Faculty of Medicine, Nursing and Health Sciences, Monash University, Melbourne, Victoria, Australia; 7 CSIRO Health and Biosecurity Business Unit, Clayton, Victoria, Australia; 8 The Australian Centre for Behavioural Research in Diabetes, Carlton, Victoria, Australia; 9 Eastern Health Clinical School, Faculty of Medicine, Nursing and Health Sciences, Monash University, Melbourne, Victoria, Australia; 10 Monash Centre for Health Research and Implementation, School of Public Health and Preventive Medicine, Monash University, Clayton, Victoria, Australia; 11 Diabetes and Endocrinology Units, Monash Health, Clayton, Victoria, Australia; 12 Institute for Health Transformation, Faculty of Health, Deakin University, Warrnambool, Victoria, Australia; 13 School of Agriculture and Food Science, University College Dublin, Dublin, Ireland; 14 SAHMRI Women and Kids, South Australian Health and Medical Research Institute, Adelaide, South Australia, Australia; 15 Health Economics and Policy Evaluation Research (HEPER) group, Faculty of Pharmacy and Pharmaceutical Sciences, Monash University, Melbourne, Victoria, Australia

**Keywords:** Diabetes in pregnancy, Pregnant Women, Protocols & guidelines, Diabetes Mellitus, Type 2

## Abstract

**Abstract:**

**Introduction:**

Women with gestational diabetes mellitus (GDM) are at seven-fold to ten-fold increased risk of type 2 diabetes mellitus (T2DM) when compared with those who experience a normoglycaemic pregnancy, and the cumulative incidence increases with the time of follow-up post birth. This protocol outlines the development and validation of a risk prediction model assessing the 5-year and 10-year risk of T2DM in women with a prior GDM diagnosis.

**Methods and analysis:**

Data from all birth mothers and registered births in Victoria and South Australia, retrospectively linked to national diabetes data and pathology laboratory data from 2008 to 2021, will be used for model development and validation of GDM to T2DM conversion. Candidate predictors will be selected considering existing literature, clinical significance and statistical association, including age, body mass index, parity, ethnicity, history of recurrent GDM, family history of T2DM and antenatal and postnatal glucose levels. Traditional statistical methods and machine learning algorithms will explore the best-performing and easily applicable prediction models. We will consider bootstrapping or K-fold cross-validation for internal model validation. If computationally difficult due to the expected large sample size, we will consider developing the model using 80% of available data and evaluating using a 20% random subset. We will consider external or temporal validation of the prediction model based on the availability of data. The prediction model’s performance will be assessed by using discrimination (area under the receiver operating characteristic curve, calibration (calibration slope, calibration intercept, calibration-in-the-large and observed-to-expected ratio), model overall fit (Brier score and Cox-Snell R2) and net benefit (decision curve analysis). To examine algorithm equity, the model’s predictive performance across ethnic groups and parity will be analysed. Transparent Reporting of a multivariable prediction model for Individual Prognosis Or Diagnosis-Artificial Intelligence (TRIPOD+AI) statements will be followed.

**Ethics and dissemination:**

Ethics approvals have been received from Deakin University Human Research Ethics Committee (2021–179); Monash Health Human Research Ethics Committee (RES-22-0000-048A); the Australian Institute of Health and Welfare (EO2022/5/1369); the Aboriginal Health Research Ethics Committee of South Australia (SA) (04-23-1056); in addition to a Site-Specific Assessment to cover the involvement of the Preventative Health SA (formerly Wellbeing SA) (2023/SSA00065). Project findings will be disseminated in peer-reviewed journals and at scientific conferences and provided to relevant stakeholders to enable the translation of research findings into population health programmes and health policy.

**STRENGTHS AND LIMITATIONS OF THIS STUDY**

- Using a large, diverse, population-based dataset, this study will develop models to predict risk of type 2 diabetes mellitus in women with prior gestational diabetes mellitus.
- Use of routinely collected administrative data enables inclusion of real-world predictors, enhancing the model’s clinical relevance and implementation potential.
- The large sample size strengthens statistical power and allows for the inclusion of multiple predictors without risking overfitting.
- As the data were not collected specifically for this study, there may be missing or unmeasured variables; a thorough evaluation of missing data and appropriate imputation methods to address these issues will be applied.

## Introduction

 Gestational diabetes mellitus (GDM) is defined as hyperglycaemia with onset during pregnancy in women without prior known type 1 or type 2 diabetes mellitus (T2DM).^1^ The increasing rates of GDM and T2DM across the globe have created a growing health and economic burden^2^,^4^ that affects around 18% of pregnancies and up to 30% in high-risk populations.^5^ This increasing prevalence of GDM aligns with rising obesity rates and advanced maternal age.^6^,^8^ It has also been attributed to wider adoption of the International Association of Diabetes in Pregnancy Study Group (IADPSG) criteria, which introduced a lower fasting plasma glucose threshold (≥ 5.1 mmol/L) or 2 hours postprandial glucose level (≥ 6.7 mmol/L) that captures a larger overlapping cohort than previous criteria.^9^,^11^

Systematic reviews and meta-analyses have identified that women with a previous diagnosis of GDM have a seven-fold to ten-fold risk of converting to T2DM compared with those who experienced a normoglycaemic pregnancy^12 13^ with the highest relative risk in the first 5 years post birth.^12^ A recent systematic review of models predicting postpartum glucose intolerance with previous GDM identified 15 studies across six different countries or regions: four from the USA, six from Europe, two from Australia and one each from Asia, Canada and Ethiopia over the period 1995–2022.^14^ Common variables in the final predictive models by these studies included body mass index, fasting glucose concentration during pregnancy, maternal age, family history of T2DM, biochemical variables (lipid metabolites, triacylglycerols and cholesterol), oral glucose tolerance test (OGTT) result during pregnancy, use of insulin during pregnancy, postnatal fasting glucose level, genetic risk factors, Haemoglobin A1c (HbA1c) and weight. Seven of the 15 studies were internally validated, but no external validation was evident.^14^ Identifying and validating key predictive variables across diverse populations is essential to improving early detection and prevention of T2DM in women with a history of GDM.

Changes to GDM diagnostic criteria have had significant implications for clinical use and diabetes prevention in Australia and internationally.^5 15^ The adoption of the IADPSG criteria^10^ and subsequent impact on GDM prevalence is yet to be fully understood, in addition to considering predictive variables and methodological approaches. While lifestyle interventions do reduce T2DM risk in women with previous GDM,^16^ engaging them in postpartum screening and prevention programmes is challenging.^17 18^ The engagement barriers exist at the personal, health provider and healthcare system levels and include time pressures, screening guideline ambiguity and changes in lifestyle attributed to caring for a newborn.^19^ The development of a risk prediction model provides an opportunity to identify risks and then develop and validate risk prediction probabilities to determine T2DM conversion risk in women with previous GDM. This model will be useful for developing and implementing targeted monitoring and prevention programmes in women most at risk of developing T2DM. It will also align with national policy (eg, Australian National Diabetes Strategy 2021–2030)^20^ and recent international efforts to better understand risk prediction in this population.^21^

Existing prediction models for T2DM in women with prior GDM rely on small, clinically focused populations. This data linkage protocol is part of a larger study (Mothers and Gestational Diabetes in Australia-2, MaGDA-2) and builds on previous research undertaken in the MAGDA study, which reported on a systems approach to prevent T2DM in women with previous GDM.^18^ By integrating routinely collected administrative health data, this study will facilitate the identification and tracking of long-term health outcomes across diverse populations, providing a cost-effective and comprehensive approach to risk prediction. This protocol expands on the development and international validation of early risk-prediction tools,^22^ including recent validation using the IADPSG diagnostic criteria^23^ and risk prediction for adverse pregnancy outcomes.^24^ Using Boyle *et al*’s data linkage framework,^17^ which linked perinatal statistical data collection from two Australian states, national diabetes data and private pathology providers, this study aims to identify 5-year and 10-year T2DM risk factors at both the population and individual level to develop and validate risk prediction models, stratified by country of birth, in women with previous GDM.

## Methods and analysis

### Study design

We will conduct a prediction model development and validation study using population-based datasets that have been retrospectively linked. Development and validation of the risk prediction model will be informed by recent developments in the field^25^,^28^ and the framework presented by Chen *et al*.^29^ We propose to develop an analogous tool that predicts post-GDM conversion to T2DM; this provides a sound framework. The findings will be reported using the recent Transparent Reporting of a multivariable prediction model for Individual Prognosis Or Diagnosis-Artificial Intelligence (TRIPOD+AI) statement guidance for reporting clinical prediction models that use regression or machine learning methods.^30^

### Participants, data sets and linkage

Study participants will include mothers of all births registered in two Australian state perinatal registers (Victoria’s Consultative Council on Obstetric and Paediatric Mortality and Morbidity, CCOPMM; South Australia’s South Australian Perinatal Statistics Collection, SAPSC), with linked data to national diabetes datasets and pathology laboratories from 2008 to 2021 inclusive. The study will use all records from the state perinatal registers (CCOPMM and SAPSC), and Diabetes Australia’s National Gestational Diabetes Register (NGDR). Non-identifiable patient data will be linked from 2008, allowing the use of existing linked data to be updated and tracked until the end of 2021 to align with the broader project end date. Variables to be captured are presented in table 1. Data acquisition and linkage methods are available in online supplemental figure 1.

**Table 1:** Data sets for linkage

Database	Description	Key variables
Consultative Council on Obstetric and Paediatric Mortality and Morbidity (CCOPMM, Victoria)	A perinatal database with records of all births and mother birth history registered in Victoria, Australia.	Demographic variables, including age, ethnicity, country of birth, indigenous status, clinical history including number of previous pregnancies, number of previous births, history of having a baby with macrosomia, gestation at birth, history of obstetric complications, glucose intolerance and family history of T2DM and anthropometric variables (body mass index (BMI)), will be extracted from records.
South Australian Perinatal Statistics Collection (SAPSC, South Australia)	A perinatal database with records of all births and mother birth history registered in South Australia, Australia.	Demographic variables, including age, ethnicity, country of birth, indigenous status, clinical history including number of previous pregnancies, number of previous births, history of having a baby with macrosomia, gestation at birth, history of obstetric complications, glucose intolerance and family history of T2DM and anthropometric variables (BMI), will be extracted from records.
Diabetes Australia National Diabetes Services Scheme	A national database with records of all persons registered with a diagnosis of T2DM.	A confirmed diagnosis of T2DM.
National Gestational Diabetes Register	A national database with records of all persons registered with a diagnosis of GDM.	A confirmed diagnosis of GDM, pharmacological treatment of GDM and a history of GDM.
Selected Victorian and South Australian pathology laboratories	Records of all antenatal, perinatal, postnatal and follow-up pathology testing for women registered on the CCOPMM or SAPSC databases.	Values for diagnostic and screening pathology tests including oral glucose tolerance test, glycated haemoglobin test, glucose challenge test and blood glucose.

GDM, gestational diabetes mellitus; T2DM, type 2 diabetes mellitus.

Pathology results obtained from participating pathology laboratories will be used to confirm T2DM diagnosis and collect ongoing T2DM-related testing as part of postpartum outcomes tracking. The CCOPMM and SAPSC data will be explored for suitable explanatory/independent variables to develop and validate risk prediction models. Data from the National Diabetes Services Scheme and NGDR will be used to confirm T2DM and GDM diagnoses. Follow-up activities initiated by the NGDR will be tracked against laboratory data of any T2DM screening activities undertaken. This approach has been used by previous research.^17^

### Eligibility criteria

All women with IADPSG criteria diagnosed with GDM during the study period will be included. Women with diabetes confirmed prior to pregnancy will be excluded. For women with GDM in a prior pregnancy recorded in the dataset, only the most recent birth will be included in the analysis, with earlier pregnancies contributing to the ‘previous history of GDM’ variable in the most recent record.

### Treatment received

Following GDM diagnosis, the guidelines^10 31^ recommend a 2-hour group lifestyle education session led by a diabetes nurse educator and dietitian (https://www.adips.org/) or online. The health behaviour changes include dietary adjustments, increased physical activity and weight management, noting that complete adherence to guidelines cannot be guaranteed. Guideline adherence data have not been collected for this study as it is not available in existing administrative datasets. Follow-up appointments with an endocrinologist or endocrinology trainee are typically scheduled every 1–3 weeks to monitor glycaemic control.^10^ Insulin therapy is initiated if predefined glucose targets are not met (fasting <5.5 mmol/L and 2-hour postprandial <7.0 mmol/L). Metformin is considered for specific cases, particularly when there is inadequate response to insulin therapy alone.^10 32^

### Outcome

The outcome to be predicted will be the 5-year and 10-year risk of developing T2DM post birth (the exact postpartum follow-up timeframe will depend on data availability and power) in women with previous GDM, with models developed for categories based on country of birth. T2DM will be diagnosed based on the WHO criteria of having any of the following:

- HbA1c level of 6.5% (48 mmol/mol) or higher.
- Fasting plasma glucose level ≥7.0 mmol/L.
- 2-hour plasma glucose level ≥11.1 mmol/L.

### Predictors

Potential predictors will be identified from a literature review of existing prediction models of T2DM after GDM^12 16 33^ and national and international screening guidelines.^34^ An initial list of candidate predictor variables was drafted (table 2) based on a multidisciplinary team consultation of study investigators working in women’s health, including obstetricians, endocrinologists, biostatisticians and consumer stakeholders (table 2).

**Table 2:** List, definition and data type of the candidate predictors

Predictor variable	Definition	Data type
Age	Age at service use	Years, continuous (no decimal point)
Height	Height of participants	Centimetres, continuous (no decimal point)
BMI	Body mass index of the participants	kg/m^2^, continuous (no decimal point)
Gestational weight gain (GWG)	GWG from preconception or early pregnancy to 36 weeks or beyond.	g
Ethnicity	Ethnicity was categorised as: Australian (not Aboriginal or Torres Strait Islander) Oceanian European Middle Eastern, North African or Sub-Saharan African Southern and Central Asian South-East and North-East Asian People of the Americas and other NEC.	Categorical data
Past history of recurrent gestational diabetes (GDM)	GDM developed during previous pregnancies	Binary
Gestational age at GDM diagnosis	Gestational age at which GDM is diagnosed	Weeks, continuous
GDM with insulin treatment	Management of GDM with insulin	Binary
Family history of diabetes (including Type 1 diabetes (T1DM), type 2 diabetes (T2DM) or GDM)	Defined as: First degree relative with history T1DM or T2DM Sister with GDM or hyperglycaemia in pregnancy	Binary
Past history of pre-eclampsia/eclampsia	Pre-eclampsia/eclampsia developed during previous pregnancies	Binary
Chronic hypertension	Hypertension diagnosed or present before pregnancy or on at least two occasions before 20 weeks of gestation.	Binary
Polycystic ovary syndrome (PCOS)	Presence of PCOS	Binary
Parity (nulliparous or parous)	The number of times a woman has given birth	Count data, categorised
Gravida	Total number of confirmed pregnancies	Count data
Past history of macrosomia	History of macrosomia in the past pregnancies, defined as: birthweight >4500 g or >90th centile	Binary
Pregnancy complications	Composite variables of pregnancy complications	Binary
Antenatal 2-hour oral glucose tolerance test (OGTT) (mmol/L)	2 hours post 75 g oral glucose load test in antenatal period	Continuous
Antenatal fasting plasma glucose (FPG)	Fasting plasma glucose test in antenatal period	Continuous
Postnatal FPG (mmol/L)	Fasting plasma glucose test in postpartum period	Continuous
Postnatal 2-hour OGTT	2 hour post 75 g oral glucose load test in postpartum period	Continuous
Breastfeeding	Duration of postnatal breastfeeding,	Months, continuous
	Breastfeeding at discharge	Category

### Sample size

This data linkage study is expected to generate a large and robust dataset suited for the development and validation of risk prediction models. Using a recommended approach to calculate the minimum required sample size, as outlined in recent guidelines,^35^,^38^ demonstrates that the study is adequately powered. This sample size calculation considers factors including the baseline outcome prevalence (T2DM, 10.3%),^39^ number of model predictor parameters to be used (up to 21 in our case), anticipated C statistic (0.71)^40^ and shrinkage (0.9). Based on these parameters, a minimum sample size of 3467 participants (with 358 events/T2DM) will be required, assuming a 0.05 acceptable difference in apparent and adjusted R-squared and 0.05 margin of error in the estimation of intercept. With 14 years of data linkage (2008–2021) from large registries in two major states, we expect a substantial dataset with >7000 women with previous GDM, exceeding existing sample size calculations. This larger sample will enhance the stability of coefficients, leading to a more robust prediction model. Internal validation does not require a separate sample. We will also consider external or temporal validation of the prediction model based on availability data. This data will not be used in the model development. To temporally and/or externally validate an existing multivariable prediction model for a binary outcome,^37^ using previous parameters and normally distributed linear predictors with a mean (standard deviation (SD)) of −2.63 (0.996), C statistic CI width (0.1) and observed/expected ratio CI width=1, the minimum sample size required for model validation is 6363 with 656 events.

### Data preparation and missing data handling

All data analyses will be conducted using STATA V.17.0 or R software V.4.4.2, as necessary. The same data preprocessing method will be carefully applied for both development and validation datasets separately, as recommended. Descriptive summaries and histograms will be used to assess continuous predictors. Anomalies such as duplicates, skewed distributions and outliers will be handled using appropriate statistical methods.

Each explanatory variable will be examined for patterns of missing values to determine whether data is missing at random or missing completely at random. The missing indicator method will be used for predictors with missing data that are not at random. For missing data at random, multiple imputation by chained equations will be applied to impute missing values with: (1) polytomous logistic regression for multilevel (>two levels) categorical and (2) binary logistic regression for dichotomous categorical variables. Based on the volume of data expected, we do not anticipate loss of power due to missing values, with imputation undertaken primarily to minimise potential bias. Validated outcomes with linkage to data sources will be used to minimise outcome ascertainment bias. We will provide a supplementary table comparing the distributions of predictor variables between participants with and without missing data. The minimum sample size required for external validation will be adjusted considering the amount of missingness. The same missing data handling method for the development and validation dataset will be considered. Predictors with more than 40% missing values will be excluded from further consideration to minimise the risk of biased estimates when data are excessively missing.^41^

### Planned statistical analyses

Descriptive statistics will be used to describe the demographic characteristics of all women, including breakdowns by those who do and do not subsequently develop T2DM post-GDM within the study timeframe for both the development and validation datasets. Characteristics of women will be compared using t-tests for continuous measures (non-parametric equivalents used where required) and χ^2^ tests for categorical variables. The same process will be undertaken to compare women diagnosed using the 1991 Australasian Diabetes in Pregnancy Society diagnostic criteria^31^ and the IADPSG ones.^10^ The linked laboratory data will facilitate this comparison.

Crude and age-standardised annual prevalence rates of GDM and those who subsequently develop T2DM post GDM will be calculated as a percentage of total annual pregnancies, using direct standardisation to the maternal age structure of the entire study population. Ethnicity groups based on self-reported ethnicity (if available) or country of birth as recorded will be explored using the following categories: Australian (excluding those flagged as Aboriginal or Torres Strait Islander), Aboriginal or Torres Strait Islander (with indicated flag), Oceanian, European, Middle Eastern, North African or Sub-Saharan African, Southern and Central Asian, South-East and North-East Asian, People of the Americas, and other (not elsewhere classified (NEC)).^23^ Prevalence rates over time will be examined by maternal age group, ethnicity and BMI. The time from GDM diagnosis (typically OGTT during pregnancy) to T2DM diagnosis (WHO definition from laboratory results) will be calculated for each case and then stratified by key explanatory variables, where available such as age, parity, ethnicity and BMI, with comparisons between subgroups. Time from GDM to T2DM diagnosis will be a key variable to calculate an individual’s risk of conversion. Other derived variables will include the presence of a GDM and T2DM screening tests. The rates of developing T2DM post GDM will be compared between all GDM cases.

### Model development

Candidate predictor variables will be selected a priori based on existing literature and clinical expertise (see table 2 for the draft list). In addition, during model development, baseline characteristics will be examined using Pearson’s χ^2^ tests and univariate logistic regressions. These exploratory analyses will use a conservative p<0.20 (after age adjustment) and variables meeting this criterion will be considered for inclusion in the initial multivariable predictive models. Variables with high clinical value (driven by expert input from study investigators) will also be investigated in the initial multivariable predictive models regardless of the p value on univariate testing. The multicollinearity of independent variables will be investigated using the variance inflation factor. Alternatively, the least absolute shrinkage and selection operator method will also be employed to select the predictors, and the results will be compared with the method described above.

Once all predictors have been selected, considering the existing literature, clinical significance and statistics, we will consider traditional statistical methods and machine learning algorithms to explore the best-performing and easily applicable prediction models for our binary outcome of developing T2DM (Yes/No). We will consider various machine learning techniques, such as the K-Nearest Neighbour classifier, Decision Tree classifier, Random Forest classifier and XGBoost classifier, and compare the model performance metrics with traditional logistic regression models. The resulting predictive performance of each model will be rigorously compared using appropriate metrics to identify the optimal approach for T2DM risk prediction for further evaluation and deployment.

### Model evaluation

Internal validation: we will consider bootstrapping or K-fold cross-validation for model internal validation. However, if this becomes computationally difficult due to the expected large sample size, we will consider developing the model using 80% of available data and evaluating it using a 20% random subset. The data are sufficiently large to split, so using a random subset for internal validation will be our pragmatic choice.^26^

Temporal validation: due to the large number of records over the proposed linkage period, we will create a separate temporal validation sample from the women who gave birth more recently. These data will not be used in the model development.

Following the recommended method of updating models, we will report performance metrics after recalibrating the model by applying correction factors to the calibration intercept, if there are any changes in the prevalence reported in model development and validation datasets.^42^

### Assessment of model performance

After selecting the optimal prediction model approach, we will use the validation dataset to generate predicted values. The predicted probabilities will be summarised using mean, SD and histograms for individuals with and without T2DM. To calculate the prediction performance metrics, we will compare these predicted values with the actual values and generate performance metrics, including discrimination, calibration and decision curve analysis. The area under the receiver operating characteristic curve (AUROC) will be used to assess the discrimination performance (the model’s ability to correctly identify those with the condition and those without). Calibration slope (the spread of predicted risk compared with observed outcomes), calibration intercept, calibration-in-the-large and observed-to-expected ratio metrics will be used to assess model calibration (the agreement between the model’s estimated risk and the observed outcome). To assess the percentage of variations in outcome explained by the model, we will use the model overall fit assessment metrics such as Brier score and Cox-Snell R2. All performance measures will be presented with 95% CIs. We will compare the apparent performance with the true performance to assess optimism from overfitting. To examine algorithm fairness (which identifies any clear biases in the algorithm),^30^ we will analyse the model’s predictive performance across categories of ethnicity and parity. We will develop models using imputed data to see the effect of missing values and compare the findings with a complete case analysis.

### Sensitivity analysis

We will conduct a separate analysis that excludes cases involving known insulin treatment to assess the confounding effect of insulin treatment on the associations between predictors and outcomes.^43^

### Model presentation

Different risk prediction model presentations will be considered depending on the selected optimal model for ease of clinical applicability. If logistic regression is selected, we may consider establishing a scoring system by converting the coefficients of the regression model into a quantifiable scoring system using appropriate mathematical operations. We will also consider a user-friendly option, such as developing websites or applications with a risk calculator. The final predictive model predicting T2DM after GDM will be presented visually in an electronic app similar to the current online personal GDM tool^44^ to facilitate translation into clinical settings and is analogous to absolute cardiovascular risk tables,^45^ which enables general practitioners to rapidly identify which patients are at high risk of a cardiovascular event and require health behaviour changes, prescribed medication(s) or both.

### Assessment of clinical utility

The decision curve analysis will assess threshold probabilities where the model net benefit is better than alternative decision strategies. This analysis evaluates the benefit of using our model to identify women at risk for T2DM who can potentially benefit from lifestyle interventions that may reduce their risk. Decision curve analysis will compare the net benefit of our model (considering true and false positives/negatives) across different risk thresholds to treating all or none of the women.

Probability (risk) thresholds including the Youden Index (sum of sensitivity and specificity)^46^ and thresholds associated with 10% false positive and false negative rates will be examined. Worked examples of population level impacts of applying these thresholds will be discussed with our expert and patient advisory groups in order to inform the utility of model implementation.

### Methodological considerations

Methodological considerations to be noted with the use of these datasets, methods of data acquisition and linkage include the inability to validate record linkage quality due to privacy protection—a limitation cited in previous data linkage research.^17^ This method was tested and validated alongside traditional mechanisms for linking person-identifiable data to ensure comparable sensitivity and specificity to verify linkage efficacy.^47^

### Patient and public involvement

Patient and public community members will be engaged as potential end users to inform the interpretation, presentation of findings and dissemination of the model. Priority populations, including Aboriginal or Torres Strait Islander communities, will be engaged in line with ethical protocols approved by the Aboriginal Health Research Ethics Committee (AHREC)(a subcommittee of the Aboriginal Health Council of South Australia). Other cultural groups at high cardiometabolic risk, such as South Asian communities, will also be engaged through culturally tailored initiatives, including partnerships with community leaders, culturally appropriate health education and multilingual resources. A consumer advisory group with representation of various priority groups will guide the project with lived-experience perspectives. Representatives from this group will be embedded in the project leadership team for ongoing input and all input will be considered in decision-making processes. Their involvement will range from consultation to active partnership, including participation in design workshops, review of study materials for cultural and linguistic appropriateness, co-development of dissemination strategies and membership in the governance and decision-making committees. This approach ensures meaningful inclusion of their voices in the study. Examples of patient and public involvement include discussing worked examples of population level impacts after applying a variety of cut-off thresholds, in order to inform the utility of the model. Research findings will be presented to relevant stakeholders, including policymakers, healthcare professionals and advocacy groups, to enable translation into policy and practice. The consumer advisory group will guide the dissemination pathway of the findings.

## Ethics and dissemination

### Data storage, retention and access

Approval for the health data linkage in Victoria and South Australia is provided under strict conditions to protect privacy and confidentiality. Current approval permits data storage on a secure server hosted by the National eResearch Collaboration Tools and Resources, a Commonwealth-funded project to develop National eResearch Collaboration Infrastructure managed through the University of Melbourne. Data governance will be overseen by the Project Board and the Project Management Group, guided by data minimisation principles, regular review of a project risk register, adherence to the data management plan and regular reporting to the project board. It is anticipated that the final data linkage will be complete in the third quarter of 2025 with formal analysis to be undertaken in the fourth quarter of 2025 and the first quarter of 2026.

### Ethics approvals

An initial ethics application for MAGDA-2 was approved by the Deakin University Human Research Ethics Committee (DUHREC) on 26 June 2021 (2021-179). To access the perinatal data in South Australia and Victoria, a National Mutual Acceptance (NMA) ethics approval was required, so the DUHREC approval was superseded by the Monash Health Human Research Ethics Committee (MHHREC) (approved on 24 June 2022 (RES-22-0000-048A)), with ongoing approval until a final progress report is submitted. For the data linkage component, the project received ethics approval from the Australian Institute of Health and Welfare (granted on 17 April 2023 (EO2022/5/1369)). A separate ethics approval was also obtained from the self-identified Aboriginal flag in the SAPSC data extraction, which was approved by the AHREC of South Australia on 30 October 2024 (04-23-1056) until 30 October 2027. The NMA MHHREC approval met the NMA requirements of South Australia (SA) Health; however, a Site-Specific Assessment (SSA) was required to cover the involvement of the Preventative Health SA (formerly Well-being SA) and to comply with SA Health policy requirements. A Site-Specific Assessment was approved on 30 October 2023 (2023/SSA00065) until 30 October 2026.

### Dissemination

Findings will be reported by the Reporting of studies Conducted using Observational Routinely collected health Data statement (RECORD)^48^ and Guidelines for Accurate and Transparent Health Estimates Reporting (GATHER),^49^ as appropriate. Project findings will be disseminated in peer-reviewed journals and at scientific conferences and provided to relevant stakeholders to enable the translation of research findings into population health programmes and health policy.

## Discussion

A prognostic risk prediction model for T2DM in women with prior GDM will be developed and validated using a large, diverse, population-based dataset. This approach will enable the development of specific risk prediction models for women with diverse countries of birth. This study will follow the best practice for model development and validation, guided by recent evidence and guidelines. The findings will be reported in line with the TRIPOD+AI statement, which will help enhance the model’s clinical acceptability and support future implementation efforts.

There is limited research on risk prediction models for T2DM in women with a history of GDM. Of the 20 studies included in the more recent systematic review and meta-analysis,^12^ only one study was undertaken in Australia.^50^ This single-site retrospective cohort study applied the Australian Diabetes Society guidelines 1998 criteria for T2DM diagnosis^51^ and found that the cumulative risk of developing T2DM increased over 15 years of follow-up and was 9.6 times greater for women with a previous diagnosis of GDM, with a cumulative risk of 25.8% at 15 years post GDM diagnosis.^50^ Importantly, a prospective longitudinal cohort study reported that 13% (11 out of 82) of Australian First Nations women diagnosed with GDM developed postpartum diabetes during 2.5 median years of follow-up.^52^ There is limited research on risk prediction models for T2DM in women with a history of GDM. Two studies explored this topic, but they were restricted by relatively small sample sizes (n<106) and a limited number of predictor variables (primarily focused on biomarkers such as microRNA and lipidomics).^53 54^ These limitations highlight the need for a data linkage study to develop a more comprehensive risk prediction model by using routinely collected predictors. There is also a need to revise the conversion rates to T2DM following a diagnosis of GDM with consideration of other risk factors.

Using routinely collected administrative data allows for a large population sample, which is recommended for risk prediction model development. This large sample size strengthens the model’s statistical power and enables consideration of more potential predictor variables without risking overfitting. It is important to note that this data was not gathered explicitly for this study. This may introduce missing data issues such as measurement of the outcome in a selected population or leaving out potential unmeasured predictors from the model. To mitigate these concerns, a thorough evaluation of missing data and appropriate imputation methods to address these issues will be applied.

There are no risk prediction models for postpartum T2DM following GDM that are currently implemented.^14^ A model previously developed had several methodological limitations, such as the included predictors often not routinely collected, being expensive, being developed using small sample sizes, showing incomplete reporting and exhibiting low overall performance.^14^ By using routinely collected, state-level administrative registered birth data from two Australian states at scale, which covers approximately 33% of the Australian population, these methodological issues can be addressed. Contemporary Australia is very ethnically diverse and capturing data on ethnicity is likely to be a better predictor than country of birth as a result. We will be able to develop predictive models that can be potentially translated into other countries within this study because of the data being collected.

The IADPSG diagnostic criteria have significantly increased the prevalence of GDM, particularly among women with milder glucose elevations, leading to a 4.1% increase in diagnoses and a 12.1% increase among Hispanic women.^55^ This shift raises concerns about unnecessary healthcare interventions and follow-up for low-risk women.^56^ In addition, it underscores the evidence gap regarding the long-term T2DM risk for women diagnosed using these criteria and whether the long-term benefits of diagnosing milder cases exist.^56^

Using routinely collected healthcare data can enhance risk prediction accuracy,^57^ helping to identify high-risk groups for targeted timely interventions, thereby improving patient outcomes and reducing related healthcare costs.^58^ However, implementing predictive models necessitates updates to regulatory frameworks ensuring ethical concerns and data privacy are addressed, ensuring equitable healthcare delivery while overcoming algorithmic bias. This study will allow direct opportunities for translation into both policy and practice. The project’s governance structure, which includes membership of crucial project partners on the Project Board (eg, policy and government representatives), will facilitate results translation.

The developed model is expected to provide an accurate forecast that supports pregnant women and clinicians, contributes to risk prediction model research and facilitates well-informed decisions for women and healthcare providers, ultimately preventing GDM progression to T2DM. These more precise estimates may reduce unnecessary intervention and potentially reduce stress among women diagnosed with GDM regarding the long-term risks of developing further metabolic disease. This is probable because we will develop a reliable model for clinical use created using a large, high-quality dataset, built on a research plan with robust statistical analysis and validated in distinct datasets obtained from different locations using readily available and minimal variables. These factors will support the effort of clinical practice integration. Once we have developed, validated and shown the clinical impact, the next step will be recommending its adoption within clinical practice guidelines for care delivery.

## Supplementary material

10.1136/bmjopen-2025-106052 online supplemental file 1
