# Supplementary material for: Developing and validating a risk prediction model for conversion to type 2 diabetes mellitus in women with a history of gestational diabetes mellitus: protocol for a population-based, data-linkage study
Source: BMJ Open. 2025 Sep 14;15(9):e106052. doi: 10.1136/bmjopen-2025-106052 (PMC12434774; doi:10.1136/bmjopen-2025-106052)
Supplement: online supplemental file 1 [file bmjopen-15-9-s001.pdf]

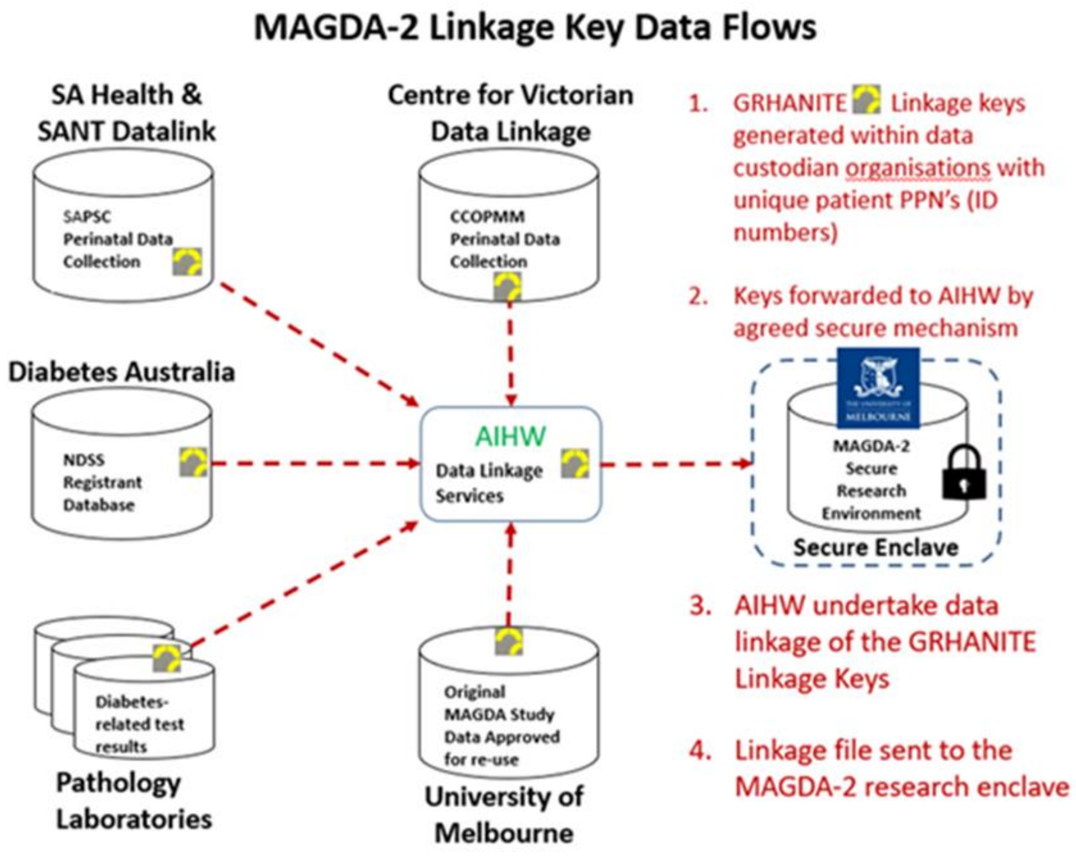

Figure 1. Data acquisition and linkage showing de-identified data flows.
