# Supplementary material for: Developing and validating a risk prediction model for conversion to type 2 diabetes mellitus in women with a history of gestational diabetes mellitus: protocol for a population-based, data-linkage study
Source: BMJ Open. 2025 Sep 14;15(9):e106052. doi: 10.1136/bmjopen-2025-106052 (PMC12434774; doi:10.1136/bmjopen-2025-106052)
Supplement: Supplementary file 2 [file bmjopen-2025-106052.reviewer_comments.pdf]

PEER REVIEW HISTORY

BMJ Open publishes all reviews undertaken for accepted manuscripts. Reviewers are asked to complete a checklist review form and are provided with free text boxes to elaborate on their assessment. These free text comments are reproduced below.

ARTICLE DETAILS

Title (Provisional)

Developing and validating a risk prediction model for conversion to type 2 diabetes mellitus in women with a previous history of gestational diabetes mellitus: protocol for a population-based, data-linkage study

Authors

Versace, Vincent; Boyle, Douglas; Janus, Edward; Dunbar, James; Feyissa, Tesfaye R.; Belsti, Yitayeh; Trinder, Peta; Enticott, Joanne; Sutton, Brett; Speight, Jane; Boyle, Jacqueline; Cooray, Shamil Deshan; Beks, Hannah; O'Reilly, Sharleen; McNamara, Kevin; Rumbold, Alice R.; Lim, Siew; Ademi , Zanfina; Teede, Helena J.

VERSION 1 - REVIEW

**Reviewer**	**1**
**Name**	**Calle-Pascual, Alfonso**
**Affiliation**	**Univ Complutense Madrid, Endocrinology and Nutrition**
**Date**	**09-Aug-2025**
**COI**	**None**

Thank you for allowing me to evaluate the protocol titled Developing and validating a risk prediction model for conversion to type 2 diabetes mellitus in women with a previous history of gestational diabetes mellitus: a data linkage protocol by Versace et al

The protocol is well designed, the data obtained comes from data-base well-identified, transparent, and recognized registries, and the planned statistical study is robust with a sample size that exceeds the expected number of events, so the predictive model for conversion to T2DM that is detected will be important.

Just a few minor comments that the authors may consider:

1. Are the authors sure that this protocol is the first predictive model? Can they guarantee it? This assertion is not usually deemed appropriate. Page 3 line 43. Especially when they acknowledge on page 5 that there are different predictive models, although they are not

validated or suffer from some methodological issue, with not well-controlled bias... Please rephrase the sentence.

2. In the identification of eligible participants, women with GDM, authors talk about nutritional treatment and physical activity during pregnancy. How will the authors analyse this?? Or is it just to recall the recommendations from the practical guide? The impression is that they will analyse it. Please explain.

3. It is well known that the onset of T2 DM in at-risk populations can be reduced with a nutritional intervention and with physical activity. Will the authors consider these modifiable factors such as postpartum lifestyle? How will they obtain and analyse this? If they are not going to analyse this, they should include a phrase in the limitations.

4. I miss planning secondary objectives. The authors do not consider that by having glucose and A1c values as continuous variables, they could estimate the predictive model for conversion to IFG, AGT Prediabetes, and/or abnormal glucose regulation?

5. I understand that they consider the onset of T2DM from normoglycemia. Do the authors consider evaluating the rates from intermediate glucose levels between normal and those diagnosed with T2DM?

6. The impression is that the predictive model only takes into account the progression from normoglycemia to T2DM. But T2DM can also regress/remit and normalize its values over time, for example, having a postpartum FPG of 10 mmol/l and then 5 mmol/L after 5 years. In another case, having a FPG of 6 mmol/L postpartum (2-4 months) with metformin and maintaining the same figures, but without treatment at 5-10 years. How would you treat these cases?

7. On the other hand, it would be very interesting to know the predictive models in a control population, that is, those who had not been diagnosed with GDM in parallel pregnancies. Would this be possible??

Otherwise, I find the protocolized study very interesting.

VERSION 1 - AUTHOR RESPONSE

Reviewer: 1	
Thank you for allowing me to evaluate the protocol titled Developing and validating a risk prediction model for conversion to type 2 diabetes mellitus in women with a previous history of gestational diabetes mellitus: a data linkage protocol by Versace et al<br><br>The protocol is well designed, the data obtained comes from data-base well-identified, transparent, and recognized registries, and the planned statistical study is robust with a sample size that exceeds the expected number of events, so the predictive model for conversion to T2DM that is detected will be important.	Thank you for taking the time to review our protocol. We appreciate your feedback and have addressed each concern separately below.
1. Are the authors sure that this protocol is the first predictive model? Can they guarantee it? This assertion is not usually deemed appropriate. Page 3 line 43. Especially when they acknowledge on page 5 that there are different predictive models, although they are not validated or suffer from some methodological issue, with not well-controlled bias... Please rephrase the sentence.	We have revised the use of 'first' as it relates to the predictive model throughout. It has been changed on Page 3 (also requested by the Editor) and the beginning of the Discussion on page 21. These revised sentences now read:<br><br>(Page 3): **Using a large, diverse, population-based dataset, this study will develop models to predict risk of type 2 diabetes mellitus in women with prior gestational diabetes mellitus.**<br><br>(Page 21): **A prognostic risk prediction model for T2DM in women with prior GDM will be developed and validated using a large, diverse, population-based dataset.**
2. In the identification of eligible participants, women with GDM, authors talk about nutritional treatment and physical activity during pregnancy. How will the authors analyse this?? Or is it just to recall the recommendations from the practical guide? The impression is that they will analyse it. Please explain.	Under the section 'Treatment received' we have included the following text to remove any ambiguity:<br><br>(Page 10): The health behaviour changes include dietary adjustments, increased physical activity, and weight management**, noting that complete adherence to guidelines cannot be guaranteed. Guideline adherence data has not been collected for this study as it is not available in existing administrative datasets.**
3. It is well known that the onset of T2 DM in at-risk populations can be reduced with a nutritional intervention and with physical activity. Will the authors consider these modifiable factors such as postpartum lifestyle? How will they obtain and analyse this? If they are not going to analyse this, they should include a phrase in the limitations.	Agreed – lifestyle interventions can be effective in reducing T2DM. The suggested analysis is beyond the scope of this study as it relates to adherence to guidelines and this data is not available at the population-level for linkage. The previously described text covers this limitation:

	(Page 10): The health behaviour changes include dietary adjustments, increased physical activity, and weight management, **noting that complete adherence to guidelines cannot be guaranteed. Guideline adherence data has not been collected for this study as it is not available in existing administrative datasets.**
4. I miss planning secondary objectives. The authors do not consider that by having glucose and A1c values as continuous variables, they could estimate the predictive model for conversion to IFG, AGT Prediabetes, and/or abnormal glucose regulation?	This suggestion is beyond the scope of the planned analysis, and we did not include formal reference to potential secondary analyses in the initial submission (as are comments 5, 6, and 7 – acknowledging their merits). We have included the following sentence in the discussion on page 24 in the 'Data statement' section:<br><br>**The focus of this protocol is the development of risk prediction models following the linkage of numerous existing datasets. With appropriate ethical considerations, beyond this primary focus, the linked dataset may be useful for numerous secondary analyses (e.g., other predictive models such as the transition to impaired fasting glucose, abnormal glucose tolerance pre-diabetes, and/or abnormal glucose regulation).**
5. I understand that they consider the onset of T2DM from normoglycemia. Do the authors consider evaluating the rates from intermediate glucose levels between normal and those diagnosed with T2DM?	As in Comment 4, this is beyond the scope of the planned analysis. As it is secondary analysis we hope the text included in the 'Data statement' section adequately covers examples of future potential for secondary analyses.<br><br>(Page 24): **The focus of this protocol is the development of risk prediction models following the linkage of numerous existing datasets. Beyond this primary focus, the linked dataset may have utility with appropriate ethical considerations for numerous secondary analyses (e.g., other predictive models such as the transition to impaired fasting glucose, abnormal glucose tolerance pre-diabetes, and/or abnormal glucose regulation).**
6. The impression is that the predictive model only takes into account the progression from normoglycemia to T2DM. But T2DM can also regress/remit and normalize its values over time, for example, having a postpartum FPG of 10 mmol/l and then 5 mmol/L after 5 years. In	This is beyond the scope of this protocol.<br>As it is secondary analysis, we hope the text included in the 'Data statement' section adequately covers examples of future potential for secondary analyses.

another case, having a FPG of 6 mmol/L postpartum (2-4 months) with metformin and maintaining the same figures, but without treatment at 5-10 years. How would you treat these cases?	(Page 24): **The focus of this protocol is the development of risk prediction models following the linkage of numerous existing datasets. Beyond this primary focus, the linked dataset may have utility with appropriate ethical considerations for numerous secondary analyses (e.g., other predictive models such as the transition to impaired fasting glucose, abnormal glucose tolerance pre-diabetes, and/or abnormal glucose regulation).**
7. On the other hand, it would be very interesting to know the predictive models in a control population, that is, those who had not been diagnosed with GDM in parallel pregnancies. Would this be possible??	What has been suggested is possible (we have all births from the state perinatal registers), but as above, this is beyond the current scope. We trust the text included in the 'Data statement' section adequately covers examples of future potential for secondary analyses.<br><br>(Page 24): **The focus of this protocol is the development of risk prediction models following the linkage of numerous existing datasets. Beyond this primary focus, the linked dataset may have utility with appropriate ethical considerations for numerous secondary analyses (e.g., other predictive models such as the transition to impaired fasting glucose, abnormal glucose tolerance pre-diabetes, and/or abnormal glucose regulation).**
